# Spatially Structured Environmental Variation Plays a Prominent Role on the Biodiversity of Freshwater Macrophytes Across China

**DOI:** 10.3389/fpls.2019.00161

**Published:** 2019-02-22

**Authors:** Min Zhang, Jorge García Molinos, Guohuan Su, Huan Zhang, Jun Xu

**Affiliations:** ^1^Freshwater Aquaculture Collaborative Innovation Center of Hubei Province, Hubei Provincial Engineering Laboratory for Pond Aquaculture, College of Fisheries, Huazhong Agricultural University, Wuhan, China; ^2^Arctic Research Center, Hokkaido University, Sapporo, Japan; ^3^Global Station for Arctic Research, Global Institution for Collaborative Research and Education, Hokkaido University, Sapporo, Japan; ^4^Division of Environmental Science Development, Graduate School of Environmental Science, Hokkaido University, Sapporo, Japan; ^5^Laboratoire Evolution et Diversité Biologique (EDB), Université de Toulouse, CNRS, ENFA, UPS, Toulouse, France; ^6^Donghu Experimental Station of Lake Ecosystems, State Key Laboratory of Freshwater Ecology and Biotechnology of China, Institute of Hydrobiology, Chinese Academy of Sciences, Wuhan, China

**Keywords:** freshwater macrophyte, functional diversity, spatial congruence, species richness, taxonomic distinctness, phylogenetic diversity

## Abstract

Different non-mutually exclusive mechanisms interactively shape large-scale diversity patterns. However, our understanding of multi-faceted diversity and their determinants in aquatic ecosystems is far from complete compared to terrestrial ones. Here, we use variation partitioning based on redundancy analysis to analyze the relative contribution of environmental and spatial variables to the patterns of phylogenetic, taxonomic, and functional diversity in macrophyte assemblages across 214 Chinese watersheds. We found extremely high spatial congruence among most aspects of biodiversity, with some important exceptions. We then used variation partitioning to estimate the proportions of variation in macrophyte biodiversity explained by environmental and spatial variables. All diversity facets were optimally explained by spatially structured environmental variables, not the pure environment effect, implying that macrophyte are taxonomically, phylogenetically, and functionally clustered in space, which might be the result of the interaction of environmental and/or evolutionary drives. We demonstrate that macrophytes might face extensive dispersal limitations across watersheds such as topography and habitat fragmentation and availability.

## Introduction

Macrophytes support many structural and functional aspects of freshwater ecosystems and their ecological properties have been extensively studied over (Westlake, 1975; Jeppesen et al., 1998; Wiegleb et al., 2015). Highly productive, macrophyte communities have a key role in carbon and nutrient fluxes, serving as sinks for organic material and sources of nutrients to the water (Jeppesen et al., 1998; Chambers et al., 2008; Hansson et al., 2010). They provide structurally complex habitat for a large diversity of organisms such as macroinvertebrate, fish and birds (Westlake, 1975; Jeppesen et al., 1998; Hansson et al., 2010). Consequently, a significant change in the structure and composition of macrophyte communities can have important knock on effects on freshwater ecosystems with important management conservation implications (Hellsten and Riihimäki, 1996; Hansson et al., 2010; Wiegleb et al., 2016; Zhang et al., 2019). The development of local macrophyte assemblages strongly depends on a variety of abiotic and biotic factors, e.g., nutrient concentrations, flow velocity, light condition, and trophic interaction (Westlake, 1975; Dodkins et al., 2005; Zhang et al., 2019). However, consensus regarding the generality of large-scale processes driving spatial variation in biodiversity of macrophytes remains elusive (Chappuis et al., 2014; Wiegleb et al., 2015).

Most macrophyte species are regarded as cosmopolitan, their broad distribution traditionally explained by common life history traits such as long-distance hydrochory, anemochory, and zoochory seed dispersal (Santamaria, 2002; Chambers et al., 2008). Such strong dispersal capacity has facilitated the evolution of their plastic response and ecological tolerances to local environmental change. For example, many macrophytes are very resilient because of their fast asexual reproduction abilities such as clonal growth and abundant propagules (Chambers et al., 2008). Consequently, under natural conditions, regional-scale taxonomic richness is generally high and relatively stable across space (Alahuhta et al., 2018). However, high environmental heterogeneity may promote species selection (environmental filtering) and persistence (niche diversification), increasing the spatial turnover of species at landscape scales (Xu et al., 2015; Alahuhta et al., 2018). Therefore, regional spatial diversity patterns in aquatic taxa may be governed by the interplay between their dispersal capacity and the spatiotemporal heterogeneity (Shmida and Wilson, 1985; Shurin, 2007; Gianuca et al., 2017; Cai et al., in press).

Three aspects of species diversity patterns (i.e., taxonomic, phylogenetic, and functional diversity) are the main focus of macroecological studies. Taxonomic diversity, the most straightforward and commonly used measurement of biodiversity in broad-scale studies (Tisseuil et al., 2013; Heino et al., 2015; Cai et al., 2018), treats all species as functionally equivalent and phylogenetically independent. However, phylogenetic diversity and functional trait variation can exert a much stronger control on biodiversity effects on ecosystem functions, such as production or nutrient cycling, than taxonomic diversity (Cadotte et al., 2011; Flynn et al., 2011). Phylogenetic diversity, incorporating evolutionary relationships between species, provides also promising way of interpreting the role of biogeographic history in community ecology (Webb et al., 2002). Therefore, studies considering all these complementary facets can provide a more complete understanding of the mechanistic links between ecological processes and evolutionary history in shaping biodiversity patterns and the provision of ecosystem services (Devictor et al., 2010; Tucker and Cadotte, 2013; Pardo et al., 2017). Here, we assess the relative contribution of spatial and environmental variables to multifaceted biodiversity patterns in macrophyte assemblages across 214 tributary drainage basins (hereafter watersheds) covering the whole China. While the existence of spatial congruence among patterns in diversity facets is an open debate, our previous work in the Yangtze River has suggested high spatial congruence of macrophyte taxonomic and functional diversity at the catchment level (Zhang et al., 2018). Whereas these patterns hold at larger, regional scales across catchments remains to be tested. On the other hand, environmental variables are themselves spatially structured (Legendre and Legendre, 2012), making it possible for spatially structured environmental variation to drive the observed variability in spatial patterns within and among diversity facets.

## Materials and Methods

### Data Acquisition and Key Definitions

Data on freshwater macrophyte species compiled from published sources (see below) was grouped at the watershed-scale (214 watersheds) across China (National Remote Sensing Center of China, as delimited by the National Council of China under the National Water Resources Strategic Plan^1^. These watersheds represent subdivisions of main river basins based on different ecohydrological criteria (e.g., river order, landscape, climate), and provide a spatial basis for the development, utilization, conservation, and management of hydrological resources in China (Cai et al., 2018). Pooling data sets into meaningful, large spatial working units such as watersheds (Pool et al., 2014) or ecoregions (Veech and Crist, 2007) is a practical, compromise solution frequently used for the analysis of macroscale diversity patterns with spatially sparse data sets (Cai et al., in press).

Although different definitions of “macrophyte” are available in the literature, we follow Cook (1974) by considering any aquatic plant that is visible to the naked eye including all higher aquatic plants, vascular cryptogams, bryophytes, and groups of algae that can be seen to be dominated by a single species. Based on this definition, we made a detailed literature review of macrophyte species in China from published (1960–2010) records related to lakes, rivers, and seasonal agricultural ponds. Documented sources included research articles and monographs together with the Scientific Database of China Plant Species^2^, the Database of Invasive Alien species in China^3^, Chinese Species Information System^4^, and gray research reports. This exhaustive literature review provided information for a total of 992 aquatic plant species. We then prepared a data matrix covering taxonomic information and functional traits of the species. To guarantee consistency across the data set we used five quality-control rules: (1) non-macrophyte species were filtered according to the Cook’s definition for macrophytes (Cook, 1974) and the records in Flora of China, (2) scientific names were standardized and synonyms were removed on the basis of the Chinese Virtual Herbarium^4^, (3) varieties were treated as a single species, (4) the distribution traits of the species were corrected according to the Flora of China, and (5) non-freshwater species were excluded. The application of these rules resulted in a total of 469 species from 214 watersheds retained for analysis, including 93 submerged species, 40 floating-leaved species, 25 free-floating species, and 311 emergent species.

### Measurements of Multiple Facets of Biodiversity

We defined taxonomic richness (TRic) as the number of species recorded in each watershed over the study period. Given the lack of sufficient and consistent phylogeny information about all included macrophytes in our data set, phylogeny diversity was assessed using taxonomic hierarchies as a proxy for phylogenetic relationships (Schweiger et al., 2008; Heino et al., 2015). Taxonomic distinctness (TDis) measures the mean taxonomic (i.e., phylogenetic) distances between species (Clarke and Warwick, 1998), which was calculated by giving equal branch lengths and six supra-species taxonomic levels (i.e., genus, family, order, subclass, class, and phylum). Hence, watershed with low TDis value indicates low phylogenetic diversity, and vice versa. TDis was calculated using the functions *“taxondive”* and *“taxa2dist”* in the R package *“vegan”* (Oksanen et al., 2016).

To calculate functional distance between macrophyte species, we gathered information on four categorical functional traits from the Flora of China, namely life form (i.e., free-floating, emergent, floating-leaved, submerged), life cycle (i.e., annual, perennial), morphology (i.e., turion, stem, rosette, leafy), sexual propagation (monoecism, dioecy), and species’ mean adult weight (Zhang et al., 2018). Morphology was defined qualitatively based on the plant description. Leafy plants typically have more lamina, often the parts concentrating the majority of photosynthesis; stem plants are those with stem and easy to propagate due to broken branches; rosette plants have a shortened stem axis and relatively large projection area that facilitates light competition; turion plants produce winter/overwintering buds as dormant storage organs in response to unfavorable ecological conditions (Cook, 1974; Adamec, 2018). Although we acknowledge that quantitative morphological traits such as shoot height, stem diameter, specific leaf area, or leaf dry mass content, available from some local studies (Fu et al., 2014, 2018), would provide a more precise assessment, these were not available given the nature of our data set and the large scale of our study area.

Four multidimensional functional diversity indices (i.e., functional richness, functional evenness, functional divergence, and functional dispersion) were computed for each assemblage. Functional richness (FRic) describes the convex hull volume filled by a community in the multidimensional functional trait space and used as a measure of the functional richness. Functional evenness (FEve) describes the evenness of the distribution of species in a community over the functional trait space by using the minimum spanning tree. Feve quantifies the regularity with which the functional space is filled by species. Functional divergence (FDiv) describes how species distribute within the volume of functional trait space. For presence/absence data, FDiv is the highest when all the species are on the convex hull and at equal distance to its center of gravity (i.e., if the center of gravity of the convex hull is also a center of symmetry of the functional space). Finally, functional dispersion (FDis) describes the mean distance of each individual species to the centroid of all other species in the assemblage (Anderson et al., 2006), which was calculated using the function *“dbFD”* in the R package *“FD”* (Laliberté et al., 2014).

### Environmental Factors

We include several major environmental factors (Supplementary Table S1) used in previous similar macroecological studies (Whittaker et al., 2001; Tisseuil et al., 2013; Cai et al., 2018), namely mean annual precipitation (MAP), mean annual temperature (MAT), solar radiation (SOLAR), and total annual runoff (RUN), as surrogates for the energy input in each watershed, together with total surface area of each watershed (AREA), and spatial variation of altitude, MAT, MAP, SOLAR, and the Shannon diversity index based on the proportions of land cover classes (forest, grass, farm, urban, water, and desert) (i.e., ALTVAR, MATVAR, MAPVAR, SOLARVAR, and LANDVAR) within each watershed representing important factors shaping biodiversity through increasing habitat diversity and availability (Whittaker et al., 2001; Tisseuil et al., 2013). ALTVAR is used as a proxy for topographic heterogeneity, calculated as the range between the maximum and minimum altitude for each watershed. The selected factors should cover the environmental drivers of macrogeographic distributions of species at the watershed scale.

All environmental data were extracted from open-access databases such as Data Sharing Infrastructure of Earth System Science, National Science and Technology Infrastructure Center^5^ and the Data Center of Institute of Geographic Sciences and Natural Resources Research, Chinese Academy of Sciences. All parameters were calculated as the average of all cell values with centroids falling within each watershed at a spatial resolution of 0.5° × 0.5° over the study period (1960–2010). Prior to the analyses, variables were logarithm or square root-transformed to improve normalization when necessary and standardized to have a mean of 0 and a variance of 1. We also computed the values of variance inflation factor (VIF) for each predictor variable before analyses to assess collinearity (Cai et al., 2018).

### Spatial Structure

Spatial structure in natural communities can be simultaneously generated by spatial autocorrelation in species assemblages and forcing (explanatory) variables, such as environmental and biotic controls or life history events (Borcard et al., 2004). In order to account for the contribution of the spatial structure of watershed configuration to observed variability in diversity facets, we used multiscale principal coordinates of neighbor matrices (PCNM; Borcard and Legendre, 2002) applied to a geographic distance matrix computed from the watershed centroid coordinates. In essence, distance between watersheds was first represented as a Euclidean distance matrix calculated from the watershed centroid coordinates. Principal coordinates analysis (PCoA) was then conducted on this matrix after truncation using a threshold distance equivalent to the minimum spanning tree of the distance matrix, defining what is considered to be “large” distances.

The resulting eigenvectors with positive eigenvalues (distance-based Moran’s Eigenvector Maps; dbMEMs), provide a spectral decomposition of any possible spatial relationships between the watersheds (Borcard and Legendre, 2002). That is, each eigenvector captures the dominant spatial structures, i.e., low-order eigenvectors (dbMEM1-3 in Supplementary Figure S1), associated with large eigenvalues, represent country-scale groupings whereas high-order eigenvectors (dbMEM69-71 in Supplementary Figure S1), associated with small eigenvalues, represent more regional-scale groupings. Following a constrained analysis, those positive eigenvectors found to significantly explain variation in macrophyte diversity were selected as independent explanatory variables for further analysis. dbMEMs were calculated using the function *“eigenmap”* with default values in the package *“codep”* in R (Dray et al., 2012).

### Data Analysis

Spatial congruence of watersheds with high diversity was assessed as the amount of concordance between the top 10% of watersheds (i.e., highest diversity values) for all six diversity indices (Pool et al., 2014). That is, the 21 watersheds with the highest FRic values (i.e., the top 10%) were compared to the 21 watersheds with the highest FEve values to calculate how many watersheds were shared within both groups. Subsequently, spatial congruence of pairwise diversity was assessed at the successive 10% intervals. A randomization procedure was performed (999 iterations) for all six diversity indices to determine whether watershed congruence was greater than the random expectations.

We used variation partitioning based on redundancy analysis (RDA) models, with significance assessed using 999 Monte Carlo permutations, to reveal the partial effects of environmental variables and spatial structure on each diversity index (Peres-Neto et al., 2006). This procedure decomposes the total variation in the response dataset into a pure spatial component (S|E), a pure environmental component (E|S), a component of the spatial structured environmental variation (E∩S) and the unexplained variation. Only significant predictor variables were used for variation partitioning as identified from multiple linear regression models by using forward selection procedure and two stopping rules: either exceeding the critical *p*-value (*P* = 0.05) or the adjusted *R*^2^ value of the reduced model against the global model based on 999 random permutations (Blanchet et al., 2008). We ran the variation partitioning analyses by using function *“varpart”* in the R package *“vegan”* (Oksanen et al., 2016). We then computed the Moran’s *I* correlograms to evaluate the degree of spatial autocorrelation of the diversity indices and the residuals from the linear models (Diniz-Filho and Bini, 2005) by using function *“correlog”* in the R package *“pgirmess”* (Giraudoux, 2016).

Given the nature of our data set, sampling bias might be expected among the 214 studied watersheds. For example, watersheds that are easy to access or largely populated may be expected to be more intensively sampled. However, Pearson correlation analysis between the number of literature sources (NOL, ranging from 4 to 23; Supplementary Figures S2a,b) compiled for each watershed, used as proxy for the total sampling effort, and species richness showed no significant correlation (Supplementary Figure S2c). Furthermore, results from a sensitivity analysis conducted by repeating the analysis only on watersheds with NOL >8 (25th quantile; *n* = 160) remain largely invariant to those obtained from the complete data set (Supplementary Figure S2d). Therefore, we report results for all watersheds.

## Results

**FIGURE 1:**
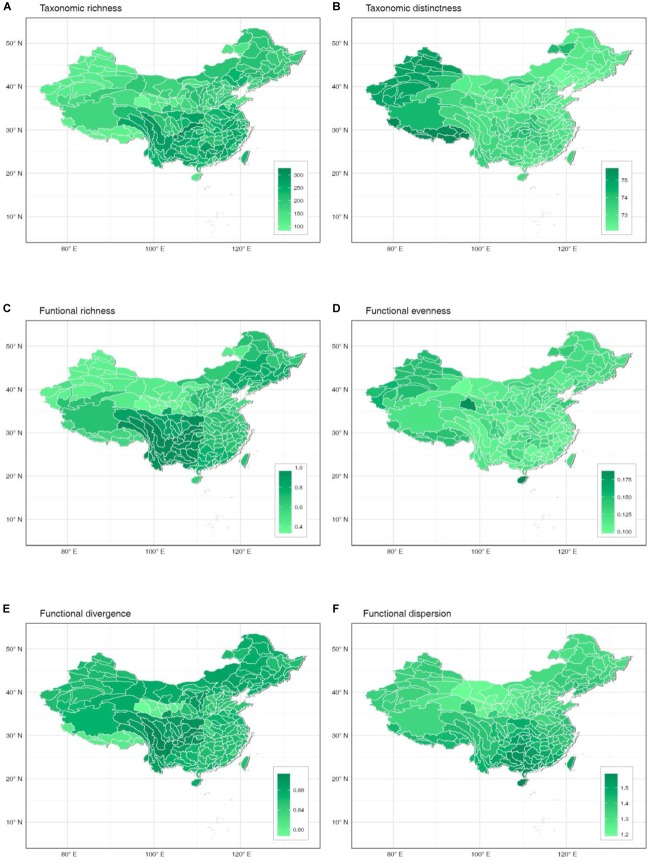
Spatial patterns of taxonomic richness **(A)**, taxonomic distinctness **(B)**, functional richness **(C)**, functional evenness **(D)**, functional divergence **(E)**, and functional dispersion **(F)** of freshwater macrophyte assemblages across Chinese watersheds.

Higher values of taxonomic richness and most functional indices concentrated in watersheds from central-southern China (Figure 1A–F). In contrast, higher values of macrophyte taxonomic distinctness and functional evenness appeared in watersheds from western China (Figure 1B–D). Among all six diversity indices, taxonomic, and functional richness presented the highest range of variation across watersheds (Figure 1A–C), with mean values representing, respectively, 62 and 70% of the total pool of 469 documented species and functional richness across China. At the other extreme, taxonomic distinctness of individual watersheds was very homogeneous across China (Figure 1B), but accounted on average for 96% of the total distinctness in the study region.

**FIGURE 2:**
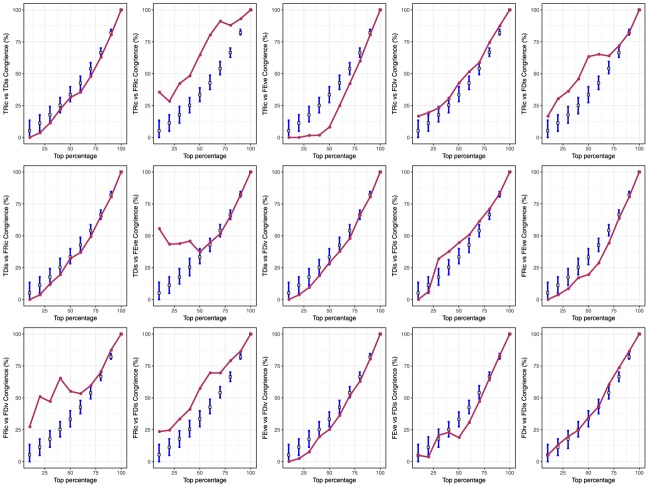
Pairwise congruence between taxonomic richness (TRic), taxonomic distinctness (TDis), functional richness (FRic), functional evenness (FEve), functional divergence (FDiv), and functional dispersion (FDis) of freshwater macrophyte assemblages across Chinese watersheds. Congruence was assessed by comparing the spatial concordance for each pair of biodiversity facets across watersheds grouped by percentiles (10% intervals).

**FIGURE 3:**
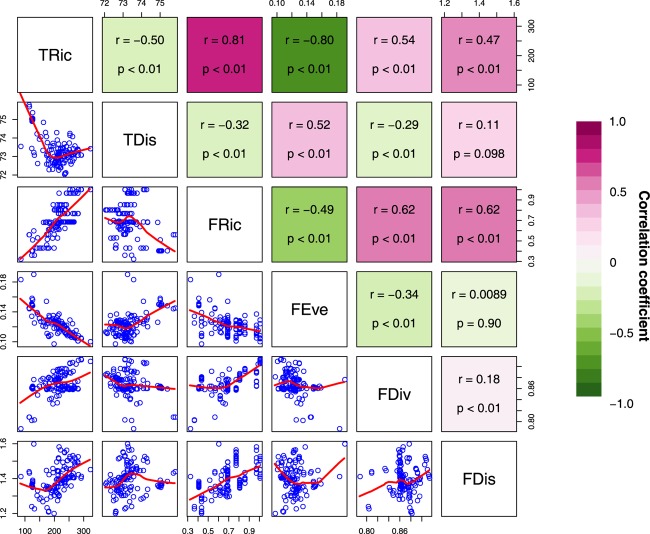
Paired scatterplots and Pearson correlations between diversity indices of freshwater macrophyte assemblages across Chinese watersheds (Abbreviations as in Figure 2). Red and blue represents positive and negative correlations, respectively.

Spatial congruence for the top 20% of each type of diversity indices presented two distinct groups with high (>40%) and low (<20%) pairwise congruence (Figure 2). The highest and lowest congruence occurred, respectively, for FDiv and FDis (58.6%) and FRic and FEve (0%). At this level, all high-congruence pairs were significantly more congruent than random expectations (999 iterations; *p* < 0.001), and showed a significant strong positive correlation (Figure 3). On the contrary, incongruent pairs showed mainly weak or strong negative (e.g., FEve – TRic and FEve – FRic) correlations (Figure 3).

**FIGURE 4:**
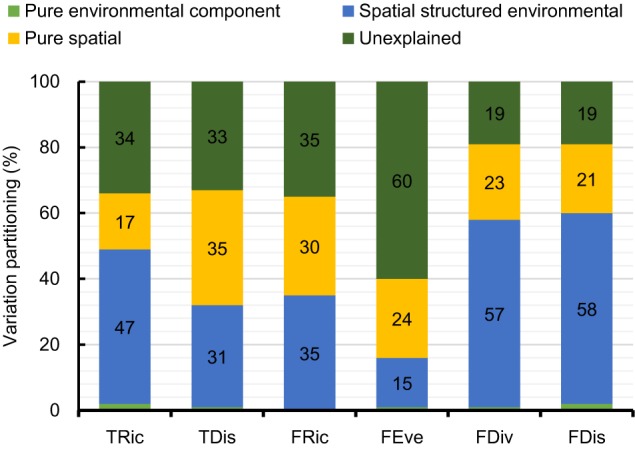
Variation partitioning for diversity indices of freshwater macrophyte assemblages across Chinese watersheds (Abbreviations as in Figure 2).

Based on the selected linear regression model interpreting the variation of the diversity indices (Table 1), the percentage of variation explained varied from 40% for FEve to 81% for FDis and FDiv (Figure 4). Within each diversity index, the proportion of variation explained by shared fractions (spatially structured environmental gradients) was significantly higher compared to the proportion by unique fractions (pure effects), although the pure spatial component was responsible for relatively high amounts of variation in TRic, FRic, FEve, and FDiv (Figure 4). Geographic variations of the six diversity indices were strongly spatially autocorrelated along with a steady decreasing of Moran’s I coefficient across distances (Supplementary Figure S3). Most of the residuals of the models showed weak spatial patterns, with the exception of the significantly positive autocorrelation at short distances, indicating that our models captured well the major ecological factors underlying geographic gradients of the diversity facets.

**Table 1:** Variables retained, adjusted R^2^, and significance values from the forward-selected multiple regression models examining the effect of environmental and spatial factors on six aspects of macrophyte biodiversity.

Functional diversity index	Factors	Selected variables	*R^2^_adj_*	*F*	*P*
Taxonomic richness	Environment	MAP + MAPVAR − ALTVAR + SOLARVAR + AREA	0.494	42.66	<0.001
	Space	dbMEM4 − dbMEM1 + dbMEM2 − dbMEM34 + dbMEM6 − dbMEM5 − dbMEM15 + dbMEM23 + dbMEM49 − dbMEM3 − dbMEM46 + dbMEM10 + dbMEM38	0.618	27.56	<0.001
Taxonomic distinctness	Environment	ALTVAR + MATVAR − MAPVAR	0.321	34.62	<0.001
	Space	-dbMEM4 + dbMEM2 + dbMEM1 + dbMEM5 + dbMEM6 − dbMEM3 + dbMEM7 + dbMEM15 + dbMEM21 + dbMEM9	0.661	42.56	<0.001
Functional richness	Environment	MAP + MAPVAR + SOLAR	0.612	110.4	<0.001
	Space	dbMEM2 + dbMEM4 − dbMEM1 − dbMEM3 + dbMEM6 + dbMEM15	0.695	81.88	<0.001
Functional evenness	Environment	MAP + ALTVAR + SOLARVAR + MAPVAR	0.331	34.62	<0.001
	Space	-dbMEM4 − dbMEM3 + dbMEM1 + dbMEM34 − dbMEM38 + dbMEM56 − dbMEM44 − dbMEM49 + dbMEM5 + dbMEM59 − dbMEM69	0.381	12.94	<0.001
Functional divergence	Environment	MAP + SOLARVAR + MAT	0.567	94.14	<0.001
	Space	dbMEM2 − dbMEM1 + dbMEM6 − dbMEM3 + dbMEM9 − dbMEM13	0.794	137.4	<0.001
Functional dispersion	Environment	MAP + MAT + SOLARVAR + SOLAR	0.901	150.3	<0.001
	Space	-dbMEM1 + dbMEM2 + dbMEM6 − dbMEM3 − dbMEM5 + dbMEM4 + dbMEM17 − dbMEM21 − dbMEM13 − dbMEM40 + dbMEM39 + dbMEM9	0.614	85.9	<0.001

*For each factor, forward selection was applied to identify which variables best described variation in functional diversity index using an inclusion threshold of alpha = 0.05.*

## Discussion

We found a relatively high spatial congruence between many, but not all, biodiversity facets (i.e., TRic and FRic, FRic and FDis, TDis and FEve, FDiv and FDis, and FRic and FDiv). Given the strong effects of spatially structured environmental factors on shaping the biodiversity patterns (Figure 1), this high spatial congruence can presumably be explained by topography-related dispersal limitation affecting specific functional groups and, consequently, species. Our results corroborate previous evidence on strong correlations between different components of diversity. For instance, Heino et al. (2008) found that species richness was highly correlated with functional richness in stream macroinvertebrate assemblages. Likewise, Strecker et al. (2011) and Pool et al. (2014) reported high spatial congruence among three aspects of species diversity patterns of freshwater fish assemblages. Meynard et al. (2011) found evidence that hypotheses generated for local and regional taxonomic diversity were equally applicable to both phylogenetic diversity and functional diversity. These findings suggest the possibility of using a single diversity measurement as a surrogate for other facets to optimize conservation planning. Given resources are often limited, pinpointing conservation priorities and simultaneously protecting multiple diversity facets is highly desirable (Devictor et al., 2010).

On the other hand, the extremely low spatial congruence found between FEve and both TRic and FRic (Figure 2), and the low spatial congruence between TDis and both TRic and FRic may be related to the definition of the measurements, whereby increases in taxonomic diversity and functional diversity can only cause small changes in phylogenetic diversity (Schweiger et al., 2008; Pool et al., 2014; Cai et al., 2018). The scatter of the negative correlation indicates that taxonomic and functional richness increase with decreasing phylogenetic and functional diversity (Figure 3). This suggests a stronger effect of species identity relative to that of taxonomic and functional richness. For instance, watersheds from central-southern China show highest TRic and FRic but relatively low FEve and TDis, whereas watersheds from western China show relatively high FEve and TDis but low TRic and FRic. Macrophytes from watersheds in central-southern China were phylogenetically and biologically more closely related, whereas those in western China were more distantly related to each other. An increasing number of studies reveal spatial mismatches among the three aspects of species diversity patterns (Heino et al., 2005; de Carvalho and Tejerina-Garro, 2015), suggesting that species occurring locally may originate from regional species pools with distinct biogeographic and evolutionary processes, respectively (Losos, 2008; Devictor et al., 2010). In other cases, species in the same region might respond differently to environmental variables affecting spatial TDis patterns and result in mismatches among different facets of diversity (Tucker and Cadotte, 2013). Thus, it is not surprising that great longitudinal gradients in China from west to east are associated with distinct environmental filtering conditions and dispersal limitations affecting functional traits and phylogenetic diversity of macrophytes.

Our results showed that both environmental and spatial factors influence the different facets of macrophyte biodiversity. In particular, spatially structured environmental gradients, rather than pure environmental effects, shaped the different facets of macrophyte biodiversity in watersheds across China (Figure 4). Pure spatial factors had a significant role in shaping several facets of the macrophyte biodiversity patterns suggesting that dispersal limitations exert a strong effect on macrophyte assemblage structure across the different diversity facets.

We found significant spatial autocorrelations among all six diversity metrics, whereas the selected spatially structured environmental variables optimally explained the spatial structure of all diversity facets. This results highlights the role of spatially structured environmental gradients, over and above the effect of environmental factors *per se*, as a major driver of biodiversity, which feeds into the debate about the effects of environmental heterogeneity and dispersal limitations on species distributions (Field et al., 2009). Our results also highlight the dominant role of climatic gradients in driving spatially structured patterns of all facets of diversity across watersheds. Climatic gradients at large spatial scales can influence biodiversity patterns through multiple mechanisms related to the physiology, energetic demand and dispersal limitations of species (Fu et al., 2018; Li et al., 2018; Zhang et al., 2019). Spatial structure in the diversity facets of TRic, FRic, FEve, and FDiv was significantly explained by broad-scale dbMEMs (Supplementary Figure S1). Macrophytes seem to generally confront dispersal limitations although they are often recognized as good dispersers (Santamaria, 2002; Chambers et al., 2008). Our findings suggest that, given the presence of mountain ranges, habitat variability, and other obstacles across the studied watersheds, macrophytes assemblages across China for most diversity aspects are strongly structured by dispersal limitation. Such a pattern of species distributions is consistent with a number of previous studies for other aquatic plant assemblages at regional scales (Capers et al., 2010; Mikulyuk et al., 2011; Alahuhta and Heino, 2013; Viana et al., 2014).

## Conclusion

Our study on variation-partitioning analysis demonstrates that macrophyte diversity patterns in watersheds across China are not always congruent and mainly driven by spatially structured environmental determinism. This finding implies that macrophyte are taxonomically, phylogenetically, and functionally clustered in space, which might be the result of environmental and/or evolutionary forces.

## Author Contributions

JX and MZ conceived the ideas and compiled the data. MZ, GS, and JX analyzed the data. MZ, JGM, GS, HZ, and JX wrote the manuscript. All authors contributed to the final manuscript.

## Conflict of Interest Statement

The authors declare that the research was conducted in the absence of any commercial or financial relationships that could be construed as a potential conflict of interest.

## Footnotes

1 http://www.nrscc.gov.cn/nrscc/

2 http://db.kib.ac.cn/eflora/Default.aspx

3 http://www.chinaias.cn/wjPart/index.aspx

4 http://db.kib.ac.cn/

5 http://zgswz.lifescience.com.cn/
