# Supplementary material for: Spatially Structured Environmental Variation Plays a Prominent Role on the Biodiversity of Freshwater Macrophytes Across China
Source: Front Plant Sci. 2019 Feb 22;10:161. doi: 10.3389/fpls.2019.00161 (PMC6396032; doi:10.3389/fpls.2019.00161)
Supplement: Supplementary file 1 [file Data_Sheet_1.pdf]

Supporting information

Title: Spatially structured environmental variation plays a prominent role on the biodiversity of freshwater macrophytes across China

Min Zhang[1], Jorge García Molinos[2,3,4], Guohuan Su[5], Huan Zhang[6], Jun Xu[6*]

[1] College of Fisheries, Huazhong Agricultural University, Freshwater Aquaculture Collaborative Innovation Center of Hubei Province, Hubei Provincial Engineering Laboratory for Pond Aquaculture, Wuhan 430070, P. R. China

Email: zhm7875@mail.hzau.edu.cn

[2] Arctic Research Center, Hokkaido University, N21W11 Sapporo, Hokkaido, Japan, 001-0021

[3] Global Station for Arctic Research, Global Institution for Collaborative Research and Education, Hokkaido University, N8W5 Sapporo, Hokkaido, Japan, 060-0810.

[4] Division of Environmental Science Development, Graduate School of Environmental Science, Hokkaido University, N10W5 Sapporo, Hokkaido, Japan, 060-0810.

Email: jorgegmolinos@arc.hokudai.ac.jp

[5] Laboratoire Evolution et Diversité Biologique (EDB), Université de Toulouse, CNRS, ENFA, UPS, Toulouse, France

Email: guohuan.su@univ-tlse3.fr

[6] Donghu Experimental Station of Lake Ecosystems, State Key Laboratory of Freshwater

Ecology and Biotechnology of China, Institute of Hydrobiology, Chinese Academy of Sciences, Wuhan, 430072, China

Email: zhanghuan@ihb.ac.cn

*** Corresponding author. Email: xujun@ihb.ac.cn**

Keywords: freshwater macrophyte, functional diversity, spatial congruence, species richness, taxonomic distinctness, phylogenetic diversity

Running-title: large-scale determinants of macrophyte diversity

Table S1. The explanatory environmental variables used for the different hypotheses.

Ecological hypotheses	Variables	Abbreviation
Energy	Mean annual temperature (℃)	MAT
	Mean annual precipitation (mm)	MAP
	Solar radiation (W ħ/m^2/day)	SOLAR
Environmental heterogeneity	Catchment area of each drainage basin (km^2)	AREA
	Altitudinal range (m)	ALTVAR
	Spatial range of MAT (℃)	MATVAR
	Spatial range of MAP (mm)	MAPVAR
	Spatial range of SOLAR (W ħ/m^2/day)	SOLARVAR
	Altitudinal range (m)	ALTVAR
	Shannon diversity index of land cover	LANDVAR
Dispersal	Moran’s eigenvector maps	MEMs

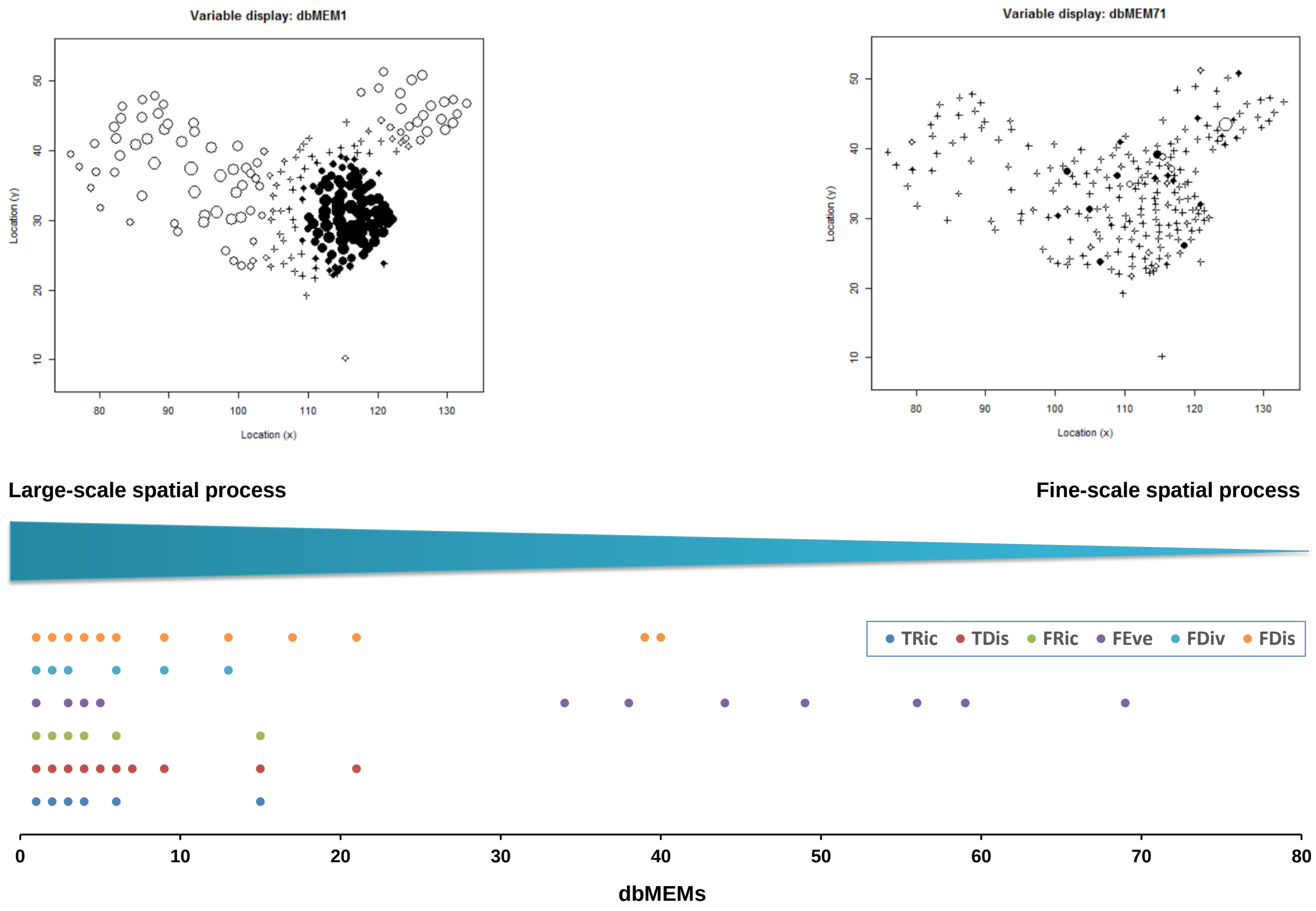

Figure S1 Selected eigenvectors (dbMEMs), based on forward selection of multiple regression models (see Methods and Table 1), for the analysis of the effects of spatial structure on each diversity facet of macrophyte lake assemblages. Top panels provide the values for the first and last eigenvectors, accounting respectively for large and small-scale spatial patterns, at the centroid of each watershed. Each symbol represents a different group as detected by the method and the size of the symbol is proportional to each watershed eigenvalue.

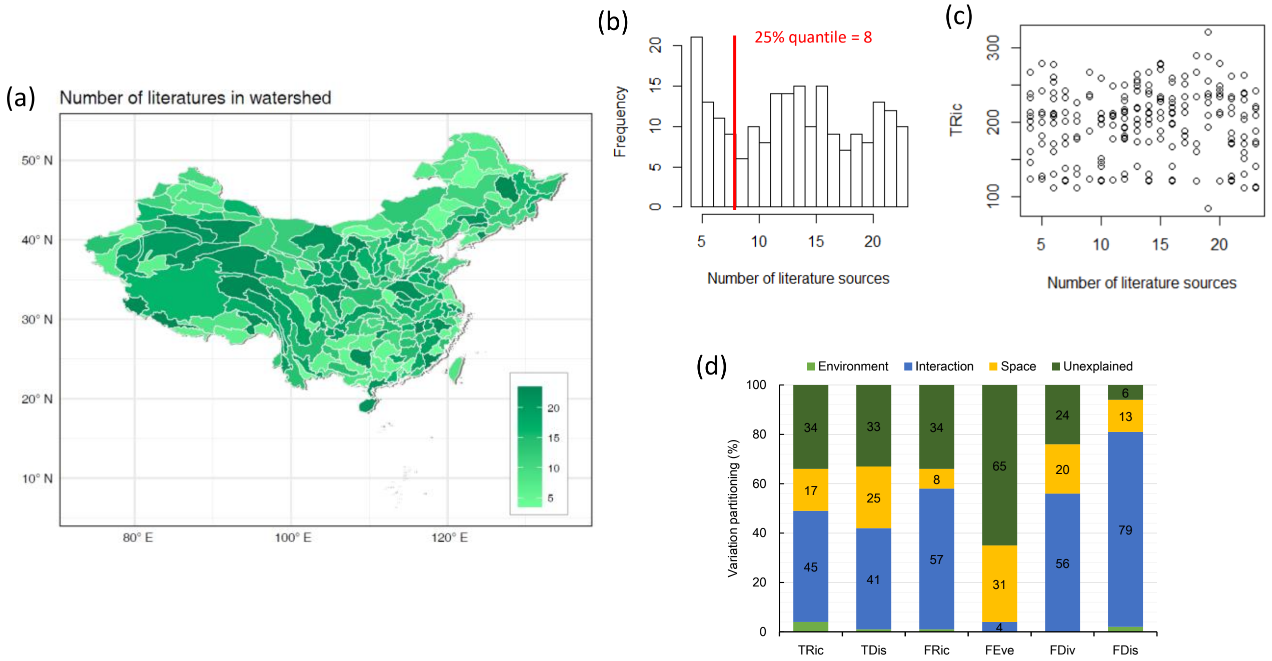

Figure S2 (a) Geographical distribution and (b) histogram of the number of literature sources compiled for each watershed with (c) its Pearson correlation with species richness. (d) Results from a sensitivity analysis conducted to test the effect of poorly sampled watersheds on the results of variation partitioning where watersheds with less than 8 sources (red line in b) were excluded from analysis.

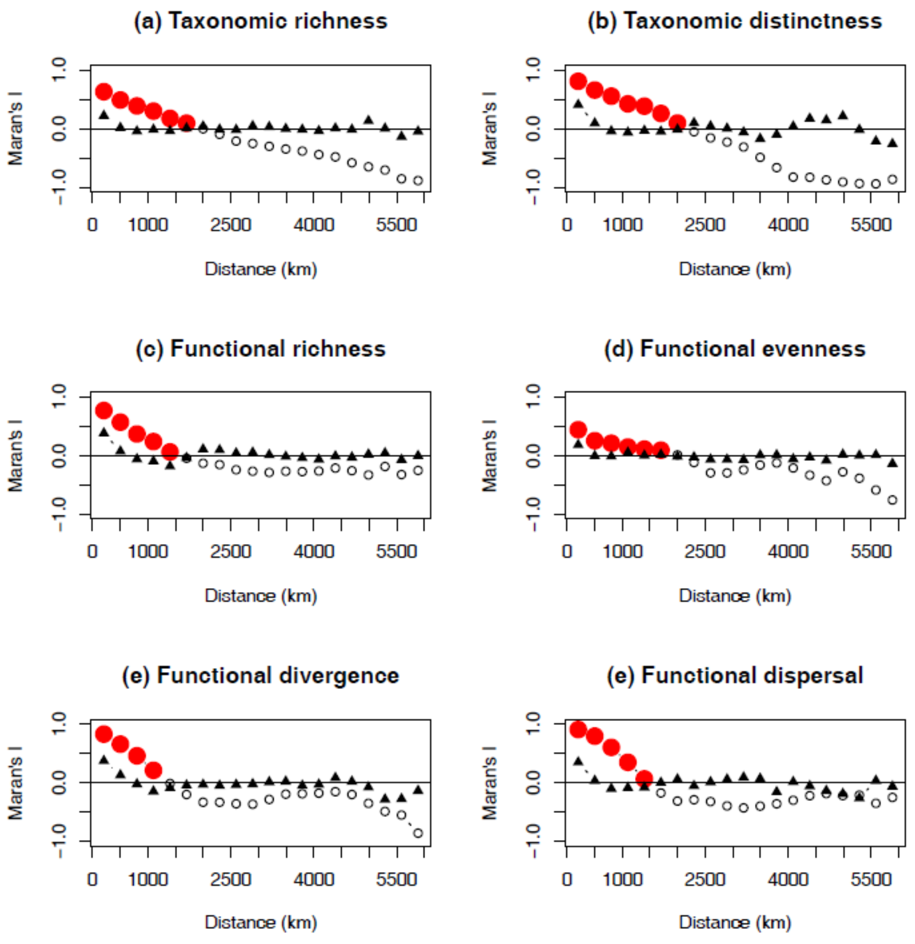

Figure S3. Spatial autocorrelation in the diversity indices (circles) and residuals (triangles) from linear regression models indicated by Moran's I statistics in relation to the geographic distances between watersheds. Moran's I statistics indicating significantly positive and negative clustered values within the distance classes are shown with filled circles.
